# A Study on Predicting Split Renal Function Using Renal Blood Flow Weighted Estimated Glomerular Filtration Rate From Arterial Spin Labeling MRI

**DOI:** 10.1002/jmri.70365

**Published:** 2026-05-21

**Authors:** Zhiyong Lin, Yuxi Li, Rui Wang, Wei Li, Jinxia Zhu, Bernd Kuehn, Jianxing Qiu, Jianping Li

**Affiliations:** ^1^ Department of Radiology Peking University First Hospital Beijing China; ^2^ Department of Cardiology Peking University First Hospital Beijing China; ^3^ Institute of Cardiovascular Disease, Peking University First Hospital Beijing China; ^4^ Hypertension Precision Diagnosis and Treatment Research Center, Peking University First Hospital Beijing China; ^5^ Information Center, Peking University First Hospital Beijing China; ^6^ MR Research Collaboration, Siemens Healthineers Ltd. Beijing China; ^7^ Research & Clinical Translation, Magnetic Resonance, Siemens Healthineers AG Erlangen Germany; ^8^ State Key Laboratory of Vascular Homeostasis and Remodeling, Peking University Beijing China

**Keywords:** arterial spin labeling MRI, glomerular filtration rate, renal artery stenosis, renal blood flow, split renal function

## Abstract

**Background:**

Traditional methods for evaluating split renal function, including contrast‐enhanced CT and radionuclide imaging, have limitations related to ionizing radiation or potentially nephrotoxic contrast agents.

**Purpose:**

To investigate the value of renal blood flow (RBF)‐weighted estimated glomerular filtration rate (eGFR) derived from arterial spin labeling (ASL)‐MRI in predicting split renal function in patients with suspected renal artery stenosis (RAS).

**Study Type:**

Retrospective.

**Population:**

Fifty‐three consecutive patients (21 females; mean age, 61.28 ± 13.50 years) with suspected RAS and renal insufficiency underwent renal ASL‐MRI.

**Field Strength/Sequence:**

1.5T; 3D pseudo‐continuous ASL technique.

**Assessment:**

RBF and regions of interest (ROI) areas of the renal cortex and entire kidney were measured on oblique coronal ASL‐MRI perfusion maps. The RBF‐weighted eGFR (RBFw eGFR) for each kidney was derived by normalizing the RBF‐area product to the bilateral total eGFR. The radionuclide GFR (rGFR) obtained from renal dynamic radionuclide imaging was used as the reference standard.

**Statistical Test:**

Paired *t*‐tests, one‐way repeated‐measures ANOVA, Bonferroni test, Pearson's correlation analysis, and receiver operating characteristic (ROC) curves were used. Statistical significance was set at *p* = 0.05.

**Results:**

The cortical and entire RBFw eGFR showed strong significant correlations with rGFR (r: 0.799 vs. 0.800). The cortical and entire RBFw eGFR had high diagnostic efficiency for differentiating severe, moderate, and mild renal insufficiency (AUC: 0.934 vs. 0.936, 0.927 vs. 0.928, and 0.873 vs. 0.865).

**Data Conclusions:**

The RBFw eGFR derived from ASL‐MRI demonstrated a significant correlation with rGFR and high diagnostic performance for differentiating split renal dysfunction grades, suggesting potential for split renal function assessment in patients with RAS.

**Evidence Level:**

3 (Nonconsecutive cohort studies, usually retrospective with an imperfectly applied reference standard).

**Technical Efficacy:**

2 (diagnostic accuracy/clinical efficacy).

## Plain Language Summary

Accurate assessment of individual kidney function is important for diagnosing and treating kidney diseases. Currently, traditional methods pose risks such as radiation exposure and the injection of contrast agents. This study aims to explore a safer and innovative MRI method for predicting individual kidney function in patients with suspected renal artery stenosis, using RBF‐weighted eGFR measurements derived from ASL‐MRI. The new MRI‐based method is highly consistent with the gold standard and can effectively evaluate the renal function of individual kidneys. It is a reliable and safer alternative and is expected to become a new tool for diagnosing kidney dysfunction.

## 1 Introduction

Renal artery stenosis (RAS) was a common cause of secondary hypertension and renal insufficiency, often led to asymmetric renal perfusion and function [1, 2]. Due to the healthy kidney's ability to compensate for the decreased function of the diseased kidney, the serum creatinine level might not increase in the early stages of the disease, which could lead to missed diagnosis and delayed opportunities for early treatment. Therefore, accurate split renal function evaluation was important for diagnosis, treatment planning, and prognosis assessment in renal insufficiency. In addition, chronic kidney disease and kidney transplantation also necessitated precise individual kidney function assessment for guiding treatment and monitoring disease [3, 4]. Traditional indicators such as serum creatinine‐based estimated glomerular filtration rate (eGFR) could not distinguish the functional status of each kidney, thereby limiting their utility in patients with asymmetric renal insufficiency [5].

Radionuclide renal dynamic imaging had long been considered the gold standard for split renal function assessment [6]. However, it involved exposure to ionizing radiation, which posed potential risks, especially in patients requiring repeated examinations [7]. Moreover, the radioactive tracers might be unsuitable for children, pregnant women, and other radiation‐sensitive populations.

Contrast‐enhanced computed tomography (CT) and contrast‐enhanced ultrasound also had limitations for renal function assessment. Contrast‐enhanced CT exposed patients to ionizing radiation, and the contrast agents used might induce nephrotoxicity and deterioration of renal function [8]. Although the ultrasound contrast agent was non‐nephrotoxic, the operator's experience and interference from abdominal organs might affect ultrasound accuracy [9]. Therefore, there was an urgent need for a non‐invasive, radiation‐free, and contrast‐agent‐independent imaging technique for accurate assessment of split renal function.

Arterial spin labeling magnetic resonance imaging (ASL‐MRI), a non‐invasive imaging modality, could be applied for quantitative assessment of renal perfusion [10]. By magnetically labeling water molecules in arterial blood as an endogenous tracer, ASL‐MRI could measure microvascular blood perfusion within the renal parenchyma without exogenous contrast agents or ionizing radiation [11, 12]. This technique has shown great potential for the assessment of renal perfusion in various renal pathologies, including RAS, diabetic nephropathy, renal transplant rejection, and other acute and chronic kidney diseases [10, 13, 14, 15, 16]. Previous studies have shown that renal blood flow (RBF) measured by ASL‐MRI has a moderate to substantial positive correlation (correlation coefficient *r* = 0.62–0.82) with glomerular filtration rate (GFR) measured by radionuclide imaging [14, 15, 16].

However, ASL‐MRI could not directly measure GFR, and further exploration was required to determine its clinical utility in split renal function prediction [17, 18, 19]. To overcome this limitation, this study proposed the concept of RBF‐weighted eGFR. The fundamental basis of this study was that renal perfusion was closely related to renal function, and a kidney with higher perfusion was likely to have a greater contribution to the overall renal function. Therefore, by calculating the proportion of the RBF of a single kidney to the total of both kidneys, and multiplying it by the eGFR, the GFR value of a single kidney could be obtained.

The primary objective of this study was to investigate the clinical value of RBF‐weighted eGFR derived from ASL‐MRI in predicting split renal function in patients with suspected RAS.

## 2 Materials and Methods

This retrospective study was approved by the Bioethics Committee of Peking University First Hospital with a waiver of written informed consent.

### 2.1 Patient Selection

From March 2023 to August 2025, 53 consecutive patients (21 females, mean age: 61.28 ± 13.50 years, age range: 26.00–87.00 years) were enrolled in this study. Inclusion criteria: (1) patient age was ≥ 18 years; (2) patients suspected of having renal artery stenosis and renal insufficiency, and who had undergone renal ASL‐MRI in our hospital; (3) patients who completed ^99m^Tc‐DTPA renal dynamic radionuclide imaging and serum creatinine examination within 1 month before the ASL‐MRI examination. Exclusion criteria: (1) renal insufficiency caused by non‐renal artery stenosis factors; (2) patients with a solitary kidney on one side; (3) patients with significant artifacts and image distortion in ASL‐MRI images that rendered evaluation and measurement impossible; (4) patients who had already undergone interventional treatment for renal artery stenosis.

### 2.2 ASL‐MRI Acquisition

All MRI data were collected on a MAGNETOM Aera 1.5T MR scanner (Siemens Healthcare, Erlangen, Germany) with an 18‐channel body coil and a 32‐channel spine coil. A prototype renal ASL sequence was employed to acquire renal perfusion data, using pseudo‐continuous arterial spin labeling (pCASL) techniques in 3D acquisition that covered both kidneys. Data acquisition was performed using a 3D Turbo Gradient Spin Echo sequence optimized for ASL measurements. A 3D acquisition technique was used in order to compensate for the intrinsically low signal‐to‐noise ratio of ASL. The ASL perfusion images were acquired in the oblique coronal direction to match the longitudinal axis of the kidney, and the scanning range included the superior, inferior, anterior and posterior borders of both kidneys, but the anterior border of the scanning range did not include the descending aorta. The labeling plane was located approximately 3–4 cm above the uppermost part of the bilateral kidneys, corresponding to the level from the lower edge of the T10 vertebra to the upper edge of the T11 vertebra, perpendicular to the aorta, and closely adjacent to the upper edge of the imaging volume. The patient breathing pattern during the ASL scan was free breathing. A 3D motion correction was used, which was performed retrospectively using 3D elastic registration. The sequence parameters for pCASL [20]: single delay, TE = 17.12 ms, TR = 5000 ms, Start TI = 3000 ms, T_1,blood_ = 1200 ms, duration of the labelling = 1500 ms, acquisition time = 5 min 5 s, FOV = 300 × 150 mm^2^, matrix = 64 × 32, slices = 12, and voxel size = 4.7 × 4.7 × 6 mm^3^.

### 2.3 Measurements of ASL‐MRI

**FIGURE 1:**
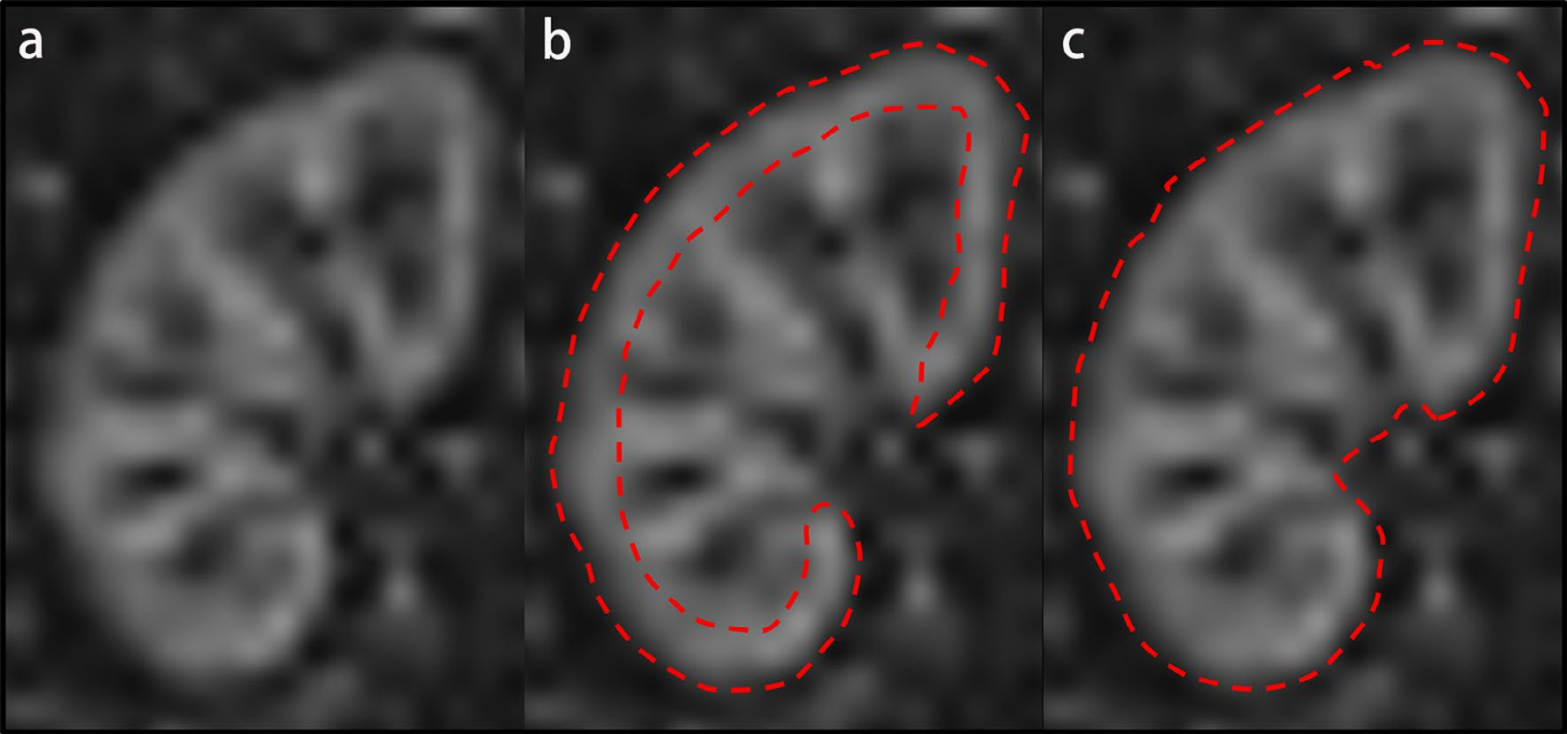
Panel (a) is the original image without regions of interest. The regions of interest used to measure RBF in the renal cortex (b) and the entire kidney (c) on ASL‐MRI images are outlined in red dashed lines. ASL‐MRI: Arterial spin labeling magnetic resonance imaging; RBF: Renal blood flow.

The RBF perfusion maps were generated inline after data acquisition. As shown in Figure 1, a reader (Z.L.: 12 years of experience in abdominal radiology) selected the three most central layers of each kidney on the oblique coronal RBF perfusion maps of pCASL MRI, and manually delineated the regions of interest (ROIs) for the renal cortex and the entire kidney respectively, thereby measuring and obtaining the corresponding RBF values and ROI area values. The average cortical RBF and the average entire RBF were obtained by averaging the RBF values of the ROIs of the renal cortex and the entire kidney, respectively. The total ROI area values of the renal cortex and the entire kidney were obtained by summing the area values of the ROIs of the renal cortex and the entire kidney at the three most central layers of each kidney, respectively.

### 2.4 Calculation of RBF Weighted eGFR

The RBF weighted cortical renal flow area product (C‐RFAP) of a single kidney is calculated by multiplying the average cortical RBF (cRBF) with the total ROI area value of the renal cortex. Similarly, the RBF weighted entire renal flow area product (E‐RFAP) of a single kidney is calculated by multiplying the average entire RBF (eRBF) with the total ROI area value of the entire kidney.

The eGFR is calculated from blood creatinine, and the cRBF weighted eGFR (cRBFw eGFR) of a single kidney is obtained by the following formula:

$$\text{cRBFw eGFR}=\text{eGFR}\times \frac{C-{\text{RFAP}}_{\text{left or right}}}{C-{\text{RFAP}}_{\text{left}}+C-{\text{RFAP}}_{\text{right}}}$$

cRBFw eGFR: cRBF weighted eGFR of a single kidney; $C-{\text{RFAP}}_{\text{left or right}}$: the value C‐RFAP of a single kidney (left or right kidney) is obtained by multiplying the average cRBF with the total ROI area value of the renal cortex; C‐RFAP_left_: the value C‐RFAP of the left kidney; C‐RFAP_right_: the value C‐RFAP of the right kidney.

The eRBF weighted eGFR (eRBFw eGFR) of a single kidney is obtained by the following formula:

$$\text{eRBFw eGFR}=\text{eGFR}\times \frac{E-{\text{RFAP}}_{\text{left or right}}}{E-{\text{RFAP}}_{\text{left}}+E-{\text{RFAP}}_{\text{right}}}$$

eRBFw eGFR: eRBF weighted eGFR of a single kidney; $E-{\text{RFAP}}_{\text{left or right}}$: the value E‐RFAP of a single kidney (left or right kidney) is obtained by multiplying the average eRBF with the total ROI area value of the entire kidney; E‐RFAP_left_: the value E‐RFAP of the left kidney; E‐RFAP_right_: the value E‐RFAP of the right kidney.

### 2.5 Renal Dysfunction Grading

According to the diagnostic criteria of the radionuclide glomerular filtration rate (rGFR) obtained from ^99m^Tc‐DTPA renal dynamic radionuclide imaging in our hospital, 106 kidneys from 53 patients were divided into four groups according to the level of renal dysfunction: normal (rGFR ≥ 34 mL/min/1.73m^2^, *n* = 17); mild (34 > rGFR ≥ 24 mL/min/1.73m^2^, *n* = 32); moderate (24 > rGFR ≥ 14 mL/min/1.73m^2^, *n* = 24); and severe (rGFR < 14 mL/min/1.73m^2^, *n* = 33). The detailed protocol for ^99m^Tc‐DTPA renal dynamic radionuclide imaging was provided in Supporting Information.

### 2.6 Statistical Analyses

Statistical analysis was performed by using statistical software (SPSS, v. 26.0; SPSS, Chicago, IL). The mean and standard deviation of RBF, renal flow area product (RFAP), RBFw eGFR, eGFR/2 (eGFR divided by 2), and rGFR parameters of single kidneys with different degrees of renal dysfunction were calculated. A paired *t*‐test was performed between cRBF and eRBF. A one‐way repeated‐measures analysis of variance (ANOVA) was conducted to compare the means of cRBFw eGFR, eRBFw eGFR, eGFR/2, and rGFR, followed by employing the Bonferroni test for pairwise comparisons and applying the Greenhouse–Geisser correction to the degrees of freedom when necessary. Pearson's correlation analysis was performed to evaluate the relationship among the RBF, RFAP, RBFw eGFR, eGFR/2, and rGFR parameters of single kidneys. Pearson's correlations ranging from 0.3 to 0.5, 0.5 to 0.7, or > 0.7 were classified as weak, moderate, and strong, respectively. Partial correlation analysis was conducted to examine the linear relationships among cRBFw eGFR, eRBFw eGFR, and rGFR, with eGFR as the control variable. The efficacy of the RBFw eGFR and eGFR/2 in the differential diagnosis of normal renal function and different levels of renal dysfunction was assessed by receiver operating characteristic (ROC) curves. Cut‐off values and areas under the curve (AUC) of the RBFw eGFR and eGFR/2 for different levels of renal dysfunction were analyzed by ROC curves. The level of statistical significance was set at *p* < 0.05.

## 3 Results

As shown in Table 1, the mean value of eRBF is slightly higher than that of cRBF (124.69 ± 42.15 vs. 119.57 ± 47.56), with a statistically significant difference (*p* < 0.001). In the repeated‐measures ANOVA conducted on the means of the cRBFw eGFR, eRBFw eGFR, eGFR/2, and rGFR, the assumptions of sphericity were violated (*p* < 0.05), so the Greenhouse–Geisser correction method was applied to adjust the degrees of freedom. The corrected results indicated that there were significant differences in the mean values of the cRBFw eGFR, eRBFw eGFR, eGFR/2, and rGFR, both in the overall group and in subgroups stratified by the level of renal dysfunction, as shown in Table 1.

**TABLE 1:** The mean and standard deviation of renal blood flow, renal flow area product, renal blood flow weighted eGFR and radionuclide GFR parameters of single kidneys with different degrees of renal dysfunction.

	Severe (*n* = 33)	Moderate (*n* = 24)	Mild (*n* = 32)	Normal (*n* = 17)	All (*N* = 106)
cRBF (ml/100 g/min)	79.27 ± 40.89	126.75 ± 42.73	135.45 ± 37.03	157.87 ± 26.96	119.57 ± 47.56
eRBF (ml/100 g/min)	85.95 ± 37.52	132.36 ± 37.15	141.42 ± 27.87	157.57 ± 21.54	124.69 ± 42.15
C‐RFAP (ml/100 g/min·cm^2^)	3846.99 ± 2515.10	8418.23 ± 3214.81	8770.62 ± 3161.63	11008.36 ± 1897.35	7516.89 ± 3816.11
E‐RFAP (ml/100 g/min·cm^2^)	7375.75 ± 4673.42	16985.49 ± 5215.11	18069.99 ± 4870.96	21722.78 ± 4311.43	15080.93 ± 7197.54
cRBFw eGFR (ml/min/1.73m^2^)	11.35 ± 7.79	23.16 ± 10.59	33.72 ± 7.72	40.35 ± 7.36	25.42 ± 13.76
eRBFw eGFR (ml/min/1.73m^2^)	11.04 ± 7.88	23.56 ± 9.83	34.09 ± 8.02	39.65 ± 7.04	25.42 ± 13.71
eGFR/2 (ml/min/1.73m^2^)	17.43 ± 9.91	21.70 ± 8.19	32.45 ± 7.74	32.73 ± 7.14	25.42 ± 10.84
rGFR (ml/min/1.73m^2^)	6.37 ± 3.13	18.90 ± 2.72	28.89 ± 3.14	41.43 ± 5.11	21.63 ± 12.88
*p* ^a^	< 0.001	0.048	0.001	0.001	< 0.001
*F* ^a^	29.359	3.483	7.364	8.198	11.387
Partial *η* ^2^ a	0.478	0.132	0.192	0.339	0.098

Abbreviations: cRBF: cortical renal blood flow; eGFR: estimated glomerular filtration rate; GFR: glomerular filtration rate; cRBFw eGFR: cortical renal blood flow weighted eGFR; C‐RFAP: cortical renal flow area product; eGFR/2: eGFR divided by 2; eRBF: entire renal blood flow; eRBFw eGFR: entire renal blood flow weighted eGFR; E‐RFAP: entire renal flow area product; rGFR: radionuclide glomerular filtration rate.

^a^ A one‐way repeated measures analysis of variance was conducted to compare the means of cRBFw eGFR, eRBFw eGFR, eGFR/2 and rGFR. The assumptions of sphericity were violated (*p* < 0.05), so the Greenhouse–Geisser correction method was applied to adjust the degrees of freedom.

**FIGURE 2:**
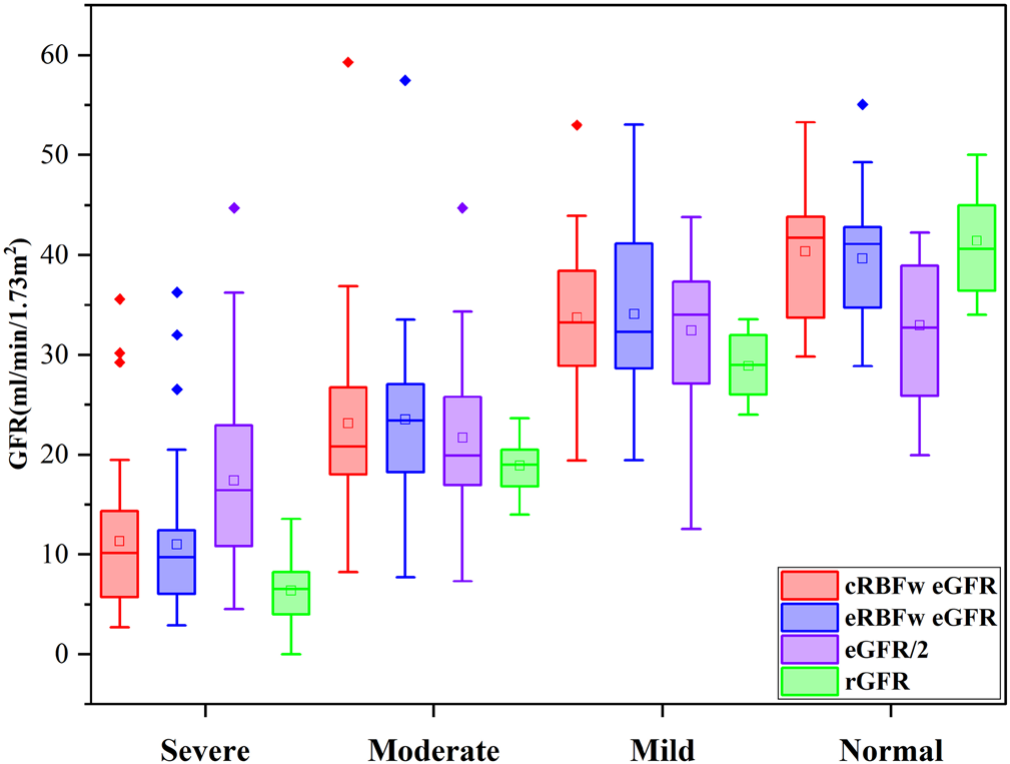
Box plots for the comparison of GFR values among cRBFw eGFR (red), eRBFw eGFR (blue), eGFR/2 (purple) and rGFR (green) in cases of normal renal function and different levels of renal dysfunction. GFR: glomerular filtration rate; eGFR: estimated glomerular filtration rate; cRBFw eGFR: cortical renal blood flow weighted eGFR; eGFR/2: eGFR divided by 2; eRBFw eGFR: entire renal blood flow weighted eGFR; rGFR: radionuclide glomerular filtration rate.

As shown in Table 2, for the overall kidneys, there was no significant difference among the mean values of cRBFw eGFR, eRBFw eGFR, and eGFR/2, whereas all these three indicators showed significant differences from rGFR. As shown in Table 2 and Figure 2, the mean values of cRBFw eGFR and eRBFw eGFR showed no significant differences among all groups stratified by the level of renal dysfunction. In the severe renal dysfunction and normal groups, the mean value of eGFR/2 showed significant differences from other indicators. In the severe renal dysfunction group, the mean values of cRBFw eGFR, eRBFw eGFR, and eGFR/2 were all significantly higher than rGFR. In the mild renal dysfunction group, the mean value of rGFR was significantly lower than that of cRBFw eGFR and eRBFw eGFR, but there was no difference compared to eGFR/2. However, in both the moderate renal dysfunction and normal groups, the mean values of cRBFw eGFR and eRBFw eGFR were not different from rGFR.

**TABLE 2:** Bonferroni test for pairwise comparisons among renal blood flow weighted eGFRs, eGFR/2, and radionuclide GFR parameters of single kidneys.

		eRBFw eGFR	eGFR/2	rGFR
Severe (*n* = 33)	cRBFw eGFR	> 0.999	< 0.001	0.001
	eRBFw eGFR	—	< 0.001	0.001
	eGFR/2	—	—	< 0.001
Moderate (*n* = 24)	cRBFw eGFR	> 0.999	> 0.999	0.339
	eRBFw eGFR	—	> 0.999	0.146
	eGFR/2	—	—	0.551
Mild (*n* = 32)	cRBFw eGFR	> 0.999	> 0.999	0.011
	eRBFw eGFR	—	> 0.999	0.008
	eGFR/2	—	—	0.081
Normal (*n* = 17)	cRBFw eGFR	> 0.999	0.006	> 0.999
	eRBFw eGFR	—	0.011	> 0.999
	eGFR/2	—	—	0.008
All (*N* = 106)	cRBFw eGFR	> 0.999	> 0.999	< 0.001
	eRBFw eGFR	—	> 0.999	< 0.001
	eGFR/2	—	—	0.002

Abbreviations: eGFR: estimated glomerular filtration rate; cRBFw eGFR: cortical renal blood flow weighted eGFR; eGFR/2: eGFR divided by 2; eRBFw eGFR: entire renal blood flow weighted eGFR; rGFR: radionuclide glomerular filtration rate.

**FIGURE 3:**
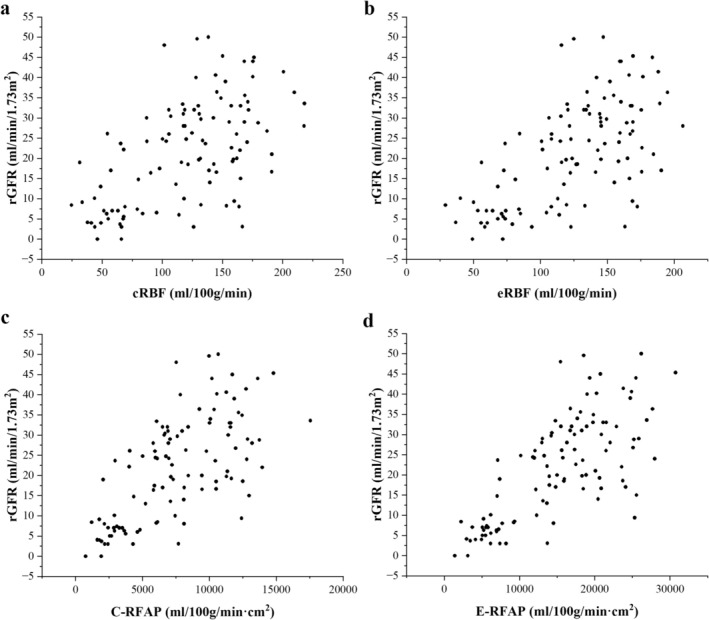
Scatter plots of the individual correlations between rGFR and cRBF (a), eRBF (b), C‐RFAP (c), and E‐RFAP (d). cRBF: cortical renal blood flow; eRBF: entire renal blood flow; C‐RFAP: cortical renal flow area product; E‐RFAP: entire renal flow area product; rGFR: radionuclide glomerular filtration rate.

**FIGURE 4:**
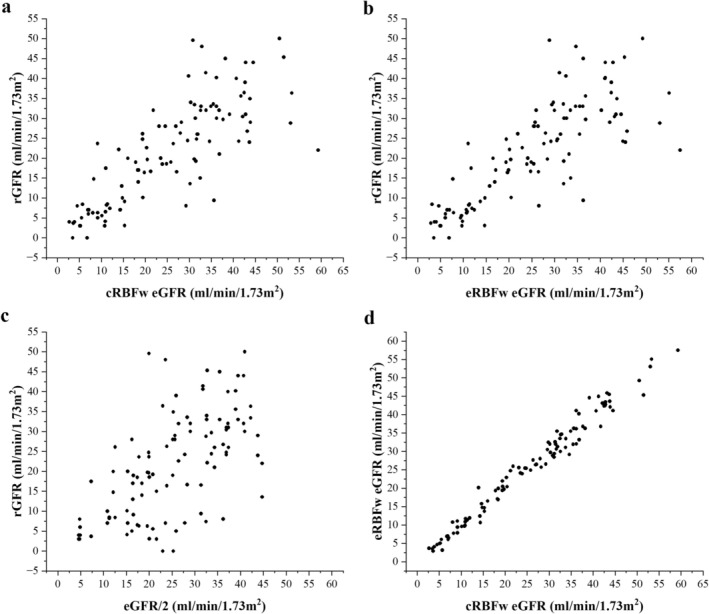
Scatter plots of the respective correlations of cRBFw eGFR (a), eRBFw eGFR (b), and eGFR/2 (c) with rGFR, along with the correlation between cRBFw eGFR and eRBFw eGFR (d). eGFR: estimated glomerular filtration rate; cRBFw eGFR: cortical renal blood flow weighted eGFR; eGFR/2: eGFR divided by 2; eRBFw eGFR: entire renal blood flow weighted eGFR; rGFR: radionuclide glomerular filtration rate.

As shown in Table 3, Figures 3 and 4, Pearson's correlation analysis showed that only E‐RFAP, cRBFw eGFR and eRBFw eGFR had strong correlations with rGFR (*r* = 0.726, 0.799, and 0.800), and the rest of the correlations with rGFR were moderate. Pearson's correlation analysis demonstrated that the RBF, RFAP, and RBFw eGFR all exhibited strong correlations between the renal cortex and the entire kidney (*r* = 0.959, 0.936, and 0.988). However, when eGFR was used as the control variable, the linear relationships between cRBFw eGFR and rGFR, and between eRBFw eGFR and rGFR were moderate, with correlation coefficients of 0.650 and 0.653, respectively. In the subgroup analysis of these 33 kidneys with severe renal dysfunction, cRBFw eGFR and eRBFw eGFR were moderately correlated with rGFR (*r* = 0.607 and 0.619), while eGFR/2 was weakly correlated with rGFR and had no statistical significance (*r* = 0.259, *p* = 0.146).

**TABLE 3:** Pearson's correlation analyses among the renal blood flow, renal flow area product, renal blood flow weighted eGFRs, eGFR/2, and radionuclide GFR parameters of single kidneys (all *p* < 0.001).

	eRBF	C‐RFAP	E‐RFAP	cRBFw eGFR	eRBFw eGFR	eGFR/2	rGFR
cRBF	0.959	0.894	0.829	0.700	0.687	0.502	0.576
eRBF	—	0.864	0.861	0.693	0.694	0.504	0.611
C‐RFAP	—	—	0.936	0.801	0.781	0.518	0.670
E‐RFAP	—	—	—	0.822	0.829	0.562	0.726
cRBFw eGFR	—	—	—	—	0.988	0.788	0.799
eRBFw eGFR	—	—	—	—	—	0.791	0.800
eGFR/2	—	—	—	—	—	—	0.613

Abbreviations: cRBF: cortical renal blood flow; eRBF: entire renal blood flow; C‐RFAP: cortical renal flow area product; E‐RFAP: entire renal flow area product; eGFR: estimated glomerular filtration rate; cRBFw eGFR: cortical renal blood flow weighted eGFR; eRBFw eGFR: entire renal blood flow weighted eGFR; eGFR/2: eGFR divided by 2; rGFR: radionuclide glomerular filtration rate.

**FIGURE 5:**
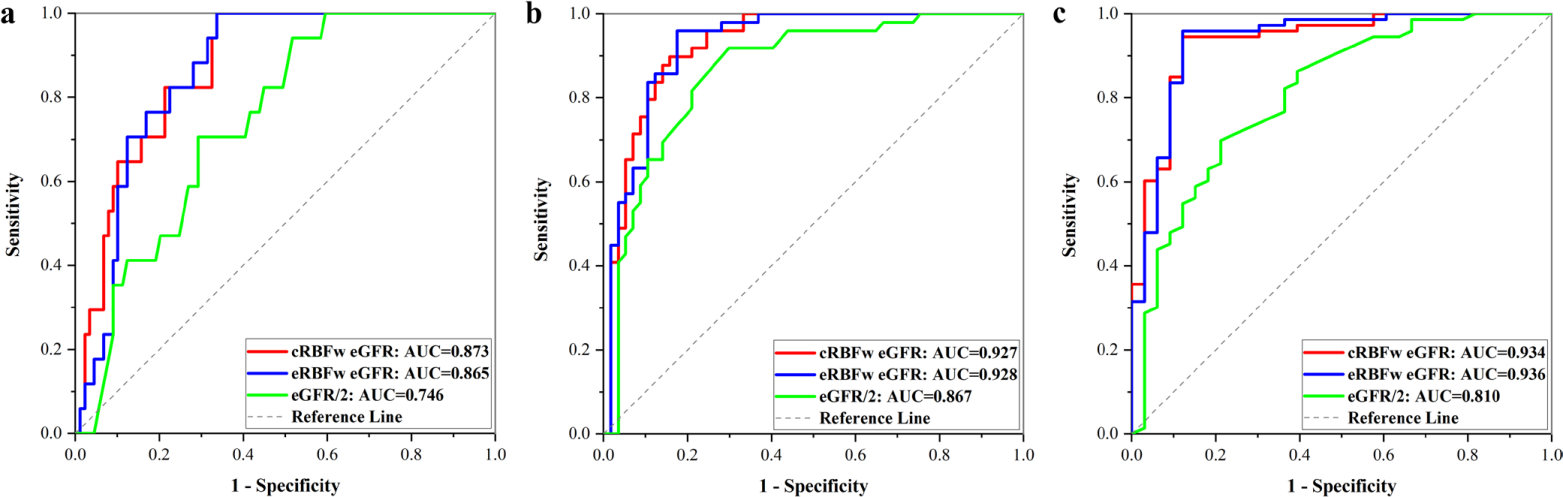
The ROC curves for the diagnosis of different grades of renal dysfunction using the RBF weighted eGFR and eGFR/2. The red line represents cRBFw eGFR, the blue line represents eRBFw eGFR, and the green line represents eGFR/2. Panels (a), (b), and (c) show the ROC curves for diagnosing mild, moderate, and severe renal dysfunction, respectively. ROC: receiver operating characteristic; RBF: renal blood flow; eGFR: estimated glomerular filtration rate; eGFR/2: eGFR divided by 2; cRBFw eGFR: cortical renal blood flow weighted eGFR; eRBFw eGFR: entire renal blood flow weighted eGFR.

As shown in the ROC curves in Figure 5, the efficacies of the cRBFw eGFR and eRBFw eGFR in the differential diagnosis of renal dysfunction were high, with an AUC range of 0.865–0.936. The AUC values of eGFR/2 were all lower than those of cRBFw eGFR and eRBFw eGFR. The cut‐off values, AUC, sensitivity, specificity, and Youden index for the indicators in the differential diagnosis of renal dysfunction were shown in Table 4. The diagnostic efficacy of cRBFw eGFR and eRBFw eGFR in the diagnosis of different levels of renal dysfunction was roughly the same. The diagnostic efficacy of eGFR/2 in the diagnosis of different levels of renal dysfunction was relatively poor compared to cRBFw eGFR and eRBFw eGFR.

**TABLE 4:** The cut‐off values, AUC, sensitivity, specificity, and Youden index for the renal blood flow weighted eGFRs and eGFR/2 of single kidneys in the differential diagnosis of renal dysfunction.

	Levels of renal dysfunction	Cut‐off value (ml/min/1.73m^2^)	AUC	Sensitivity	Specificity	Youden index
cRBFw eGFR	Severe	15.65	0.934	0.945	0.879	0.824
	Moderate	26.65	0.927	0.898	0.842	0.740
	Mild	29.73	0.873	1.000	0.663	0.663
eRBFw eGFR	Severe	16.15	0.936	0.959	0.879	0.838
	Moderate	25.43	0.928	0.959	0.825	0.784
	Mild	28.65	0.865	1.000	0.663	0.663
eGFR/2	Severe	23.71	0.810	0.699	0.788	0.487
	Moderate	22.29	0.867	0.918	0.702	0.620
	Mild	22.29	0.746	0.941	0.483	0.424

Abbreviations: AUC: areas under the curve; eGFR: estimated glomerular filtration rate; eGFR/2: eGFR divided by 2; cRBFw eGFR: cortical renal blood flow weighted eGFR; eRBFw eGFR: entire renal blood flow weighted eGFR.

## 4 Discussion

The present study demonstrates the potential of ASL‐MRI in evaluating split renal function, particularly in patients with suspected RAS. The RBFw eGFR of a single kidney based on ASL‐MRI not only shows a strong correlation with the gold standard rGFR of ^99m^Tc‐DTPA renal dynamic radionuclide imaging, but also demonstrates high diagnostic efficiency in the diagnosis of various grades of renal insufficiency. This suggests that ASL‐MRI, as a non‐invasive and radiation‐free imaging technique, could provide valuable insights into individual renal function, thus representing a promising method for the assessment of renal function.

In a previous study, ASL‐MRI was used to measure renal cortical RBF and found a correlation between RBF and decreased GFR in the early stage of diabetic nephropathy [15]. In this study, the correlation coefficient r between RBF and rGFR was 0.576–0.611, which was similar to the previous study. Our study also found that the correlation coefficient of rGFR with the product of RBF and the area of ROI was higher than that with RBF alone. This indicated that the product of RBF and the area of ROI, which represents the size of the kidney, could accurately reflect renal function.

The results showed that both cRBFw eGFR and eRBFw eGFR had a strong correlation with rGFR, and these correlations were higher than that of eGFR/2. Even when eGFR was used as a control variable, both cRBFw eGFR and eRBFw eGFR had a moderate correlation with rGFR. This indicates that combining RBF from ASL‐MRI and eGFR to calculate single‐kidney GFR was feasible, and that this method holds high clinical application value.

In the subgroup analysis of severe renal dysfunction, there was a moderate correlation between RBFw eGFRs and rGFR. Additionally, compared with rGFR, RBFw eGFRs would overestimate the eGFR value of a single kidney. Correspondingly, in the normal group, RBFw eGFRs showed no difference from rGFR. This might indicate that the RBF values of the kidney with severe renal dysfunction in this study have been overestimated.

One of the key strengths of this study lay in the comparison of two different RBF measurement methods: cRBF, which measured the renal cortical ROI, and eRBF, which measured the entire renal ROI. Interestingly, the mean value of eRBF was slightly higher than that of cRBF, while there was no significant difference between the mean values of cRBFw eGFR and eRBFw eGFR. Notably, cRBFw eGFR and eRBFw eGFR had approximately the same diagnostic efficacy for different degrees of renal dysfunction. This indicates that whether using the renal cortex or the entire kidney as the ROI, the RBFw eGFR could accurately reflect individual renal function. This suggests that there was no significant difference in the assessment of renal insufficiency between the two RBF measurement methods with different ROIs.

In this study, ROC curve analysis showed that RBFw eGFR had a high diagnostic efficiency in differentiating different degrees of renal insufficiency (AUC values ranged from 0.865 to 0.936), which was superior to eGFR/2 without RBF weighting. This further highlighted its clinical application potential. Ma et al. revealed that the RBF diagnostic model exhibited favorable diagnostic efficacy in evaluating and stratifying renal allograft dysfunction, with AUC values ranging from 0.75 to 0.96 [21], which was similar to the results of our study. Additionally, it was discovered that diagnostic models based on multi‐parameter functional MRI, such as those combining RBF with fractional anisotropy, 24‐h urinary protein, and eGFR, could enhance diagnostic performance [21]. Other studies on renal function MRI also discovered that the diagnostic model constructed by combining RBF with other indexes such as eGFR demonstrated high diagnostic efficiency in differentiating various degrees of renal dysfunction, which was significantly superior to that of a single‐index model [22, 23]. The aforementioned studies all substantiated that the approach of RBFw eGFR, which was constructed through the combination of RBF and eGFR in this study, exhibited broad applicability in the diagnosis of renal insufficiency and held high potential for clinical application.

Furthermore, this study underscores the advantages of ASL‐MRI over other renal perfusion imaging techniques. Unlike contrast‐enhanced CT, which has radiation and contrast agents with nephrotoxicity [8], ASL‐MRI is radiation‐free, non‐invasive, and does not necessitate the use of contrast agents, thereby circumventing potential nephrotoxic effects. While contrast‐enhanced ultrasonography is susceptible to operator‐dependent variability and interference from abdominal gas or adipose tissue [9], which may compromise diagnostic accuracy, ASL‐MRI could provide objective, quantitative perfusion measurements and reproducibility. Additionally, although renal radionuclide imaging using ^99m^Tc‐DTPA is able to quantify the split GFR of a single kidney, it requires intravenous nuclide injection, which is invasive and radioactive. In contrast, ASL‐MRI is suitable for routine follow‐up examinations of renal function, making it a more patient‐friendly option for patients who desire non‐invasive and radiation‐free approaches.

Although there is no need to assess split renal function for a single transplanted kidney, ASL‐MRI can be used to evaluate the blood perfusion of the transplanted kidney. ASL‐MRI can be used for long‐term monitoring of the transplanted kidney, which may facilitate early indication of potential complications such as rejection reactions or vascular problems. This early detection allows for timely intervention and management, thereby potentially improving the long‐term prognosis of renal transplant patients.

### 4.1 Limitations

Firstly, this study is only a cross‐sectional study without follow‐up, which limits our ability to assess the long‐term prognostic value of RBFw eGFR. Secondly, the generalizability of our findings to other renal diseases remains to be explored. Future studies should investigate whether the ability of ASL‐MRI technology to reflect renal function in RAS patients can be extended to the evaluation of renal function in other conditions, such as diabetic nephropathy or renal transplant rejection. Thirdly, in this study, patient scanning was performed exclusively using a 1.5T MRI system. Given that there are differences in the measurement results of RBF between 3T and 1.5T MRI devices, it is necessary to conduct further research using a 3T MRI system to verify and expand the current research findings. Fourthly, in this study, we only used a single post‐labeling delay time of 1.5 s. However, this method may lead to inaccuracies in the measurement of renal blood flow. To address this limitation, we plan to adopt the multi‐delay ASL technique in future studies, combined with the apparent transit time of blood perfusion.

## 5 Conclusion

The RBF‐weighted eGFR of single kidneys based on ASL‐MRI has a strong correlation with the rGFR determined by radionuclide renal dynamic imaging. The RBFw eGFR in the differential diagnosis of split renal dysfunction has high diagnostic efficacy. These findings indicate that the RBFw eGFR based on ASL‐MRI may have high clinical value for the diagnosis of patients with renal artery stenosis and renal insufficiency.

## Funding

National High‐Level Hospital Clinical Research Funding (High Quality Clinical Research Project of Peking University First Hospital, 2022CR77) and National High‐Level Hospital Clinical Research Funding (Research Achievement Transformation Project of Peking University First Hospital, 2022CX07).

## Supporting information


**Data S1:** jmri70365‐sup‐0001‐Supinfo.docx.
