# Supplementary material for: A Study on Predicting Split Renal Function Using Renal Blood Flow Weighted Estimated Glomerular Filtration Rate From Arterial Spin Labeling MRI
Source: J Magn Reson Imaging. 2026 May 21;64(3):712–21. doi: 10.1002/jmri.70365 (PMC13466595; doi:10.1002/jmri.70365)
Supplement: Supplementary file 1 [file JMRI-64-712-s001.docx]

***Supplementary Material 1.***

**Detailed Protocol for ^99m^Tc-DTPA renal dynamic radionuclide imaging**

1. Imaging modality

Single-photon emission computed tomography (SPECT) was utilized for renal dynamic imaging.

2. Radiopharmaceutical

The radiotracer employed was the glomerular filtration-type agent technetium-99m diethylenetriaminepentaacetic acid (⁹⁹^m^Tc-DTPA), with a radiochemical purity exceeding 95%. The administered activity ranged from 148 to 222 MBq (4–6 mCi), diluted to a total volume of 1.0 mL with sterile saline.

3. Patient Preparation:

All patients maintained normal oral intake of food and water. Thirty minutes prior to imaging, patients were instructed to ingest 300-500 mL of water and to void their bladder immediately before the examination.

4. Imaging protocol

4.1 Patient Positioning

The subject was seated upright with their back firmly positioned against the collimator. The imaging field of view (FOV) was centered over the bilateral renal regions and urinary bladder.

4.2 Radiotracer administration and dynamic acquisition

A bolus intravenous injection of 1.0 mL ⁹⁹^m^Tc-DTPA (148–222 MBq) was administered, followed by immediate initiation of continuous dynamic SPECT acquisition.

Acquisition parameters:

- Collimator: low-energy general-purpose
- Matrix size: 64 × 64
- Fast dynamic phase (renal perfusion): 2 seconds/frame × 30 frames
- Slow dynamic phase (glomerular filtration/excretion): 60 seconds/frame × 20 frames

5. Post-Processing and GFR Calculation

On the second slow dynamic phase (2–3 minutes post-injection) images, region-of-interest (ROI) analysis was performed to delineate bilateral kidneys and background regions. The renal uptake fraction was calculated, and time-activity curves (renograms) were generated for both kidneys. These curves depicted the transit of ⁹⁹^m^Tc-DTPA through the kidneys, enabling calculation of the raw glomerular filtration rate (GFR) via the Gate’s method.

The raw GFR was standardized to a reference body surface area (BSA) of 1.73 m² using the formula: Standardized GFR (mL/min/1.73 m²) = (GFR × 1.73) / Patient’s BSA, where BSA was calculated either by the Du Bois formula [BSA = 0.007184 × Height (cm)^0.725^ × Weight (kg)^0.425^] or a validated Chinese population-specific equation [BSA = 0.0061 × Height (cm) + 0.0124 × Weight (kg) − 0.0099].
